# The care pathway: concepts and theories: an introduction

**DOI:** 10.5334/ijic.812

**Published:** 2012-09-18

**Authors:** Guus Schrijvers, Arjan van Hoorn, Nicolette Huiskes

**Affiliations:** Julius Centre for Patient Oriented Research, The University Medical Centre Utrecht, The Netherlands; Programme Manager Commercial Treatment Center (CBC), The University Medical Centre Utrecht, The Netherlands; Medical Doctor Policy and Advice, CZ Health Insurance Company, The Netherlands

**Keywords:** integrated care, care pathway, clinical pathway, theory, research, evaluation, Kaiser Permanente, Cleveland Clinic, Six Sigma, business process, UMC Utrecht

## Abstract

This article addresses first the definition of a (care) pathway, and then follows a description of theories since the 1950s. It ends with a discussion of theoretical advantages and disadvantages of care pathways for patients and professionals. The objective of this paper is to provide a theoretical base for empirical studies on care pathways.

The knowledge for this chapter is based on several books on pathways, which we found by searching in the digital encyclopedia Wikipedia. Although this is not usual in scientific publications, this method was used because books are not searchable by databases as Pubmed. From 2005, we performed a literature search on *Pubmed* and other literature databases, and with the keywords integrated care pathway, clinical pathway, critical pathway, theory, research, and evaluation. One of the inspirational sources was the website of the European Pathway Association (EPA) and its journal *International Journal of Care Pathways*. The authors visited several sites for this paper. These are mentioned as illustration of a concept or theory. Most of them have English websites with more information. The URLs of these websites are not mentioned in this paper as a reference, because the content of them changes fast, sometimes every day.

## Definition of the term pathway

In 2007, Vanhaecht et al. [1] defined the term ‘care pathway’ or ‘pathway’ as follows:

“A care pathway is a complex intervention for the mutual decision-making and organisation of care processes for a well-defined group of patients during a well-defined period.

Defining characteristics of care pathways include:

- an explicit statement of the goals and key elements of care based on evidence, best practice, and patients’ expectations and their characteristics;
- the facilitation of the communication among the team members and with patients and families;
- the coordination of the care process by coordinating the roles and sequencing the activities of the multidisciplinary care team, the patients and their relatives;
- the documentation, monitoring, and evaluation of variances and outcomes, and
- the identification of the appropriate resources.

The aim of a care pathway is to enhance the quality of care across the continuum by improving risk-adjusted patient outcomes, promoting patient safety, increasing patient satisfaction, and optimizing the use of resources.”

This paper is based on this authoritative definition from the European Pathway Association (EPA) with the following comments. The expression ‘care pathway’ is leading, not the words “integrated care pathway”, ‘clinical pathway’ or ‘care street’. The term “integrated care pathway” is longer than necessary. Care pathways (as defined above) are per definition integrated. Fragmented care pathways cannot exist. The term ‘clinical pathway’ is reserved for the path within a clinic, or a 24-hour department of a hospital. A care pathway is longer and includes outpatient department’s activities, discharge from the hospital and after-care. A transmural pathway or disease management pathway [2] is even longer and also includes the preliminary and the follow-up process in primary care or other care facility.

The term ‘care street’ is a pathway within a specific architectural setting. If the latter follows and facilitates the “sequencing activities of the Multidisciplinary Team” (see definition above), then it is a care street. Good examples of care streets are seen at the Eye Hospital Rotterdam, the Coxa Hospital for hip and knee patients in the Finnish city of Tampere and the Shouldice Hernia Centre at Thornhill, Canada, which annually treats 7000 hernia patients. If a hospital or hospital division focuses on certain patient groups such as the examples mentioned here, it is a focused factory, a term that Skinner introduced in 1974. A traditional name is a categorial hospital for, for instance, rehabilitation or epilepsy. The annex of the Reinier de Graaf Hospital in The Hague in the Netherlands is an example of a focused factory designed exclusively for elective, not complicated and not-emergent interventions.

The concept of a care pathway is one of the concepts from the field of Health Operations Management (Health OM), described by Vissers and Beech as the analysis, design, planning and control of all the steps necessary to provide a service to a client [3]. They distinguish five levels of Health OM:

1. a care plan for each individual patient (patient planning and protocol);
2. the planning of care in care pathways (patient group planning and control);
3. the capacity planning of professionals, equipment and space (resource planning and control);
4. the planning of the number of patients to be treated and care activities to be carried out (patient volume planning and control), and
5. the long-term policy of the institution (strategic planning).

They emphasize the connection between these five levels. They point out the expected difficulties in introducing care pathways without taking into account any changes in scheduling systems for individual patients (level 1) and for example the capacity planning of the number of professionals and equipment (level 3). And if a pathway increases the number of patients entering the system, this will lead to decision on level 4 and 5.

Vissers and Beech distinguish two types of logistic management of care processes:

1. managing care units, and
2. the management of care processes, for instance with care pathways.

A care unit can be a health centre, an outpatient clinic, a day care centre, a laboratory, a radiology department, an operating room or a nursing ward. In such a unit logistic management is aimed at optimizing the occupancy rate of professionals and equipment. For professionals, for example, it is about avoiding gaps in the appointment schedule and about equipment such as a CT scan not being used. When managing care processes in institutions, it is important to optimize the total flow time, or the total amount of time a patient is under treatment within the institution. One approach can contradict the other if the care pathway planning (see level 2 above) and the capacity planning are not geared to one another.

### The scientific basis of the care pathway concept

Although the use of care pathways seems new for health services, the thoughts on this already exist for decades within the (defence) industry. In the 1950s, the critical path method [4] (CPM) became popular there. The first step in the CPM is to list the earliest starting date, the anticipated duration and the average degree of parallelism of all project activities to be performed. Parallelism stands for the degree to which an activity can take place simultaneously with other activities. If that is not the case and there is a logically necessary sequence, then the logical sequence will be indicated. The second step is to set up a flowchart with circles showing the start and end dates and arrows showing the activity and duration.

This diagram shows the starting point (circle one) as well as the end (circle 2) of a care pathway in an emergency department. The arrow in between indicates an activity (e.g. triage) that lasts up to five minutes.

Connecting all activities within a project shows how the duration of a project can be shortened by starting activities sooner or increasing parallelism. In later years, the graphical display of care pathways has become an art in itself. Mould et al. provide a fascinating overview of this [5]. The CPM led to the later PERT method (Program and Evaluation Technique and Review), treating statistically probable durations together with confidence intervals. This variance analysis leads to three different kinds of durations: accidental, avoidable and unavoidable time differences. In the 1980s, the company Motorola has further elaborated statistical approach from CPM and PERT into Six Sigma: a quality management approach to improve operational performances of an organization by identifying weaknesses and improving processes within the organization [6]. Literally, Six Sigma is defined as an error standard, where sigma represents the standard deviation of the average. With a value of 6σ (sigma) the number of errors does not exceed 3.4 per million (units) products or services. For example, at 4σ the number of errors is 6200 per million chances—so much higher. In that sense, the value of 6σ symbolizes the struggle for (near) perfection of the work.

The Six Sigma approach has five steps that together form the word DMAIC:

- Define: defining the problem and coordinating the project organisation;
- Measure: making the process performance concrete, i.e. a baseline run;
- Analyse: underpinning the causes of the problem;
- Improve: re-designing and optimizing the work process;
- Control: implementing and securing the improvements.

The method is particularly suited for high volume standard processes and possibilities for precise result measurement. The benefits of Six Sigma occur in the way bottlenecks and results can be interpreted in figures. In the 1990s, Six Sigma spread all over the world thanks to its success with large US companies. Since 2008, the UMC Groningen and the Reinier de Graaf Hospital are two of the hospitals in the Netherlands working with Six Sigma.

Parallel to the development of Six Sigma, Toyota managed to become one of the ten largest companies in the world thanks to ‘lean production’. And thanks to its lean production focused on creating value, the company reduced the intermediate supply and the waiting times for the activities within the factory. The Just-In-Time (JIT) concept is a part of this management philosophy. Another concept that fits in is COUP or the “Customer Order Uncoupling Point”: the point where the customer order requirements penetrate a company. Before COUP, purchase and production are based on stock. After COUP, they are based on the customer order. Translated into care pathways, this means, on the one hand, a planning without the professional actually knowing the particular patient and, on the other hand, a planning for specific patient needs. The advantage of a ‘lean’ management philosophy is that several techniques can be combined.

In the 1980s, Toyota combined its approach with Six Sigma from Motorola. Vissers and Beech [3] mention this approach as being an inviting prospect for hospital care.

### Business process redesign

In the 1990s, besides toyotism, the concept of business process redesign (BPR) came up. This was started by the articles of the Americans Hammer [7] and Davenport and Short [8]. They define a production process or business process as: the logic organisation of people, materials, energy, equipment and procedures into work activities designed to produce a specified end result (work product). They identify two aspects of these processes:

1. They have customers, either within or outside the company; and
2. They run throughout the organisation and often go beyond department limits.

Davenport and Short also emphasize the importance of information technology to design these processes and to track whether they keep running the way they were designed to. For the redesign of processes, Davenport and Short mention four goals:

1. cost reduction;
2. flow rate reduction;
3. improvement of the quality of service; and
4. improvement of job satisfaction.

Their articles had an enormous impact on corporate America. Many articles and books relied on BPR. American and Canadian Hospitals introduced BPR in the 1990s [9].

### The Theory of Constraints

Another development related to CPM and PERT is the Theory of Constraints (ToC). The Israeli business consultant Goldratt has presented this theory in 1986 in his book *The Goal* [10]. The ToC comes down to the fact that in any process bottlenecks occur that must be passed before the process can be continued. This creates a ceiling on the capacity of a system. The bottleneck in the system often can be solved with simple means. With limited investment it is often possible to make a large improvement for the overall process. Goldratt says that one hour won in dealing with the bottleneck is one hour won for the overall process. One hour spent on a non-bottleneck is a lost effort. The Theory of Constraints is based on the idea that there is at least one limiting process, assuming that the goal is set.

Goldratt distinguishes five focusing steps:

- Identify the limiting factors (people, equipment or policies ensuring that no more of the target can be achieved).
- Decide how the limiting process is used (ensuring that the limiting process does not waste time doing things that it should not do).
- Ensure that all processes are adapted to the limiting process (making all other processes subject to the restrictions and arrange them so that the limiting factor is optimally used).
- Expand the limiting process (if possible and necessary—permanent increase of capacity).
- If, as a result of these steps, the bottleneck has moved, start over from step 1.

The five focusing steps are aimed at continuous improvement around the bottlenecks. In ToC literature this is known as the Process of Ongoing Improvement (POOGI). In 1216 (!) pages the ToC Handbook [11] published in June 2010 describes many applications, some of them in healthcare. Designing pathways and ToC may be combined. In the Netherlands, the Antonius Hospital in Nieuwegein and the Sint Franciscus Hospital in Rotterdam are among those that use the ToC.

### Care pathways and health services research

The past ten years, care pathways were given a boost in the Netherlands because of the creation of multidisciplinary guidelines, on which they can be based. A good example are the integrated cancer care pathways, established in 2009 and based on the in previous years established multidisciplinary guidelines for doctors, nurses and other professionals [12]. Although the number of care pathways grows, Sermeus and Rosendal warn that not all hospital care can be placed under this heading, for a care pathway is intended for a “well-defined patient group” and a “well-defined period of time”. In patients with multiple pathology, comorbidity, severe multiple organ disease or rare disorders, there is often a lack of clear definitions at a group level and/or multidisciplinary guidelines. Also, the duration of care is difficult or impossible to predict. Sermeus estimates that 60% of hospital care can be grasped in care pathways [13]. In the Dutch mental health care, the term ‘stepped care’ is in use [14]. According to the principle of stepped care, a patient initially receives the most effective, least invasive, least expensive and shortest form of treatment that is possible given the nature and severity of the problem. After each step the next step is considered. There is not one specific care pathway here. The problem of a well-defined patient group and a well-defined period of time some authors solve by a broader definition of care pathways [15]. They distinguish fixed time care pathways (e.g. inguinal hernia surgery) and non-fixed time but phase-oriented care pathways (e.g. oncological care).

### Care pathways and care innovations

In recent years, health care providers in the Netherlands developed many new care pathways. They are process innovations that focus on improving the organization of care processes. These innovations can be distinguished from product innovations like new, scientifically proven diagnostics, treatment with drugs and equipment. Sometimes a product innovation necessitates a process innovation, for example, if oral rather than intravenous drug therapy becomes possible by a new drug entering the market. Process innovations also differ from system innovations, with different legislation, financing and legal responsibilities. Sometimes a new care pathway is not possible, because a system innovation is not realized simultaneously. If, for example, an emergency physician is not authorized by law to admit a patient (as is the case in the Netherlands) but first has to ask a surgeon for permission, a new care pathway will not lead to a shorter time of stay for patients in the emergency department.

Nowadays, in addition to new care pathways four other process innovations are also counting, namely the specialized outpatient clinic, the one-stop shop, task transfer and telecare. The specialized outpatient clinic (cf. the aforementioned focused factory), is visited by patients with similar care needs and different diseases. This clinic has a multidisciplinary character. A good example is the Falls Clinic, where patients are seen after a fall by, for example, a geriatrician and a nurse and if necessary by a neurologist, ophthalmologist or orthopaedic surgeon. A good example of a Fall Clinic is the Kennemer Gasthuis in Haarlem in the Netherlands. When this outpatient care takes place in one single day, it is the aforementioned one-stop-shop. Both the specialized outpatient clinic and the one-stop-shop usually require the design of care pathways to, within and from these units.

Task transfer from, for example, a general practitioner to a nurse practitioner or from a cardiologist to a physician's assistant is also a process innovation. The care pathways to be developed often play a major role in achieving a division of tasks between initially present professionals and newcomers. Telecare as a process innovation is to replace a face-to-face contact between patient and professional by a contact via the internet, where both parties are separated in time and space. In this case the website could take over parts of the interaction by autonomously asking questions and draw preliminary conclusions based on the responses of the patient.

Telecare also requires the design of a pathway with:

1. a division of tasks between the patient, the website and the professional;
2. easy transition from a remote contact to a face-to-face contact and vice versa
3. a maximum flow time for answering e-mail or other messages, e.g. four hours.

### Differences and similarities between pathways

Care pathways differ because patients, flow times and guidelines differ. Furthermore, there is a difference between acute and elective care pathways. The last one differs from emergency care, since the starting time of care can be planned and for common interventions the duration can be planned too, within reasonable reliability margins. There are hospitals that year after year show stable waiting times for procedures that can be planned, such as cataract and bypass surgery. Although over time, there is an increase in capacity of professionals, rooms and equipment, the access time remains a few weeks. If so, a newly developed care pathway, in combination with a catch-up effort, can help.

In emergency care this is less often the case. The starting time of care is difficult to plan, it is often hardly or not postponable and duration of elective care can also vary enormously. If a provider offers both elective and emergent care, the first mentioned care could still be delayed due to the priority of an emergency patient. With the differences between emergent care and elective care it is theoretically interesting to offer two separate care pathways for both developments within separate units. The law of large numbers makes it appealing to concentrate all emergent (surgical, internal, cardiological and so on) care in one department, the EAU (Emergent Admission Unit). In five Dutch hospitals such EAUs have since been set up, all working with their own emergent care pathways.

## Theoretical advantages and disadvantages of care pathways for patients, professionals and others

The concept of care pathways has its roots in the above-mentioned management theories: Critical Path Method, Lean Six Sigma, Business Process Redesign and the Theory of Constraints.

These theories mention the following general theoretical advantages:

1. Shortening the duration of the production process by reducing waiting time between divisions of the same organization and by simultaneously running sub-processes that take place analogously (especially, Business Process Redesign and Theory of Constraints);
2. The increase in coherence due to explicit analysis of the relationships and interactions between departments that are involved in the production;
3. Reducing the risk of errors (particularly Lean Six Sigma);
4. The reduction of the cost of the production process through standardization, by avoiding employee waiting times and underutilization of equipment, and by avoiding duplication (all theories);
5. Increasing the job satisfaction of employees as job descriptions and responsibilities derived from the work process become clearer (especially Business Process Redesign). Clarity within the set framework offers more autonomy, allowing employees to start a routine act independently without waiting for the approval of superiors.

Each of the above management theories yielded substantial savings for large companies as shown to us by the cited references. But some disadvantages are mentioned too:

- The dehumanisation of work because employees rarely have room for own creativity, for the whole production process is set up in phases and provided with a maximum duration;
- The increase in costs because data collection on the production process and control of errors and defects are expensive;
- Reduction of job satisfaction because employees do not get enough time for their activity and have no extra time to relieve stress;
- Reduction of job satisfaction because the variation in work decreases.

Theoretical advantages of care pathways for patients and professionals:

Do the mentioned advantages also apply to patients and health professionals, who are involved in the care pathway?

Advantage 1 is shortening the duration of the production process with faster diagnosis by parallelization of sub-processes every patient benefits. The earlier the diagnosis is established, the sooner treatment can begin and the shorter the period of uncertainty without diagnosis. If the treatment time is also shortened, a patient will recover faster.

Advantage 2 is the increased coherence. Greater consistency of care between different professionals provides a better overview for the patient, reduces the risk of opposing opinions and therapies, and increases the opportunity for patient empowerment.

Advantage 3 is reducing the risk of errors. Naturally, the reduced chance of errors in diagnosis and treatment works in favour of the patient.

Advantage 4 is the reduction of costs. Avoiding duplication (e.g. re-obtaining information from the patient, repetition of the same blood tests and re-entering personal information) is also in favour of patients, professionals and others. Shortening hospitalization and reducing the number of outpatient visits lead to reduction of costs.

The last advantage is related to increasing job satisfaction. When frameworks and protocols are clearly defined and coordinated between occupational groups, there is more room for freedom of action. With this increased autonomy, a nurse can start acting independently and work ahead. Dedicated, passionate professionals provide better care for the patient.

Care pathways also have theoretical disadvantages as mentioned here above. The first disadvantage is the dehumanization of work. The relationship between the health professional and the patient is less personal, the care pathway reduces the patient's choices. Furthermore, a maximum time for each patient may compromise the quality of care. Because of these time limits some care providers in Holland compare nursing according to a care pathway with stopwatch nursing and with wash streets for cars. The second disadvantage is an increase in costs. Checking for errors and defects is a costly activity and brings the professional in the position of being controlled, which could easily turn into mistrust. Furthermore, the control of errors and defects may lead to more limited access to the care pathway for patients with poor physical condition. For these patients have greater risk of, for example, postoperative infections, and a higher mortality risk. The third disadvantage is that a care pathway may lead to lower job satisfaction. If professionals get too little time to prepare and too little contact time within the care pathway, this reduces the probability of a correct diagnosis and treatment, and increases the likelihood of poor communication between patient and professional. It diminishes the dedicateness and passion of professionals. The last disadvantage is the decline of diversity in the professional work. If experienced professionals rely too much on routine, because they work within one single care pathway and always see the same types of patients, there is a chance of increasing indifference, cynicism and reduced empathy with the patient.

So far the advantages and disadvantages of care pathway as deducted for general theories.

## Discussion

In the 1950s and 1990s of the last century, the mentioned Critical Path Method, Six Sigma, Lean, Business Process Redesign, and the Theory of Constraints led to a shift compared with the paradigm of Taylor's scientific management (based on preproduction and subsequent costing of products and services) and compared with the bureaucracy model of Max Weber (which is based on hierarchical structures, clearly divided responsibilities for each unit, and with the same rules for all employees) [16]. Care pathways in Dutch health services are developed on the basis of these new paradigms, with a constant focus on the core process of the organization. From 1970 onwards, a focus is developing in the healthcare research on the statistical and business-like approach to health care processes or Health Operations Management. In the course of time, a variety of formats and concepts developed, the creation of multidisciplinary guidelines provided a huge boost for the design of care pathways.

It is not surprising that there is a period of approximately fifty years between the scientific development of the Critical Path Method and the present innovative pathways in health services. In the history of science, there are often decades between discovery (e.g. the steam engine in 1705 by the British Newcomen and Calley) and innovation (e.g. the first commercial steamship in 1809 by the American Fulton). In the U.S. health care there appears to be a period of seventeen years between the discovery of a drug or other innovation and its application in health care [17]. For care pathways, appropriate guidance and information became available only in recent years.

Applying care pathways in health services may lead to advantages and disadvantages for patients and health professionals. For managers and policy-makers this implies that their effort should be focused on maximizing the advantages and minimizing the disadvantages. For (empirical) researchers this implies that they use the right definitions to describe care pathway within a context of integrated care, disease management and health care innovation. The list of advantages and disadvantages of care pathways in this paper could be used as a checklist in empirical evaluation studies.

## Reviewers

**John Bowers,** Professor of Management Science, Stirling Management School, University of Stirling, UK

**Jan Swinkels,** MD, PhD, Psychiatrist, Professor in Clinical Guideline Development in Health Care, Academic Medical Centre, PO Box 22660,1100 DD Amsterdam, The Netherlands

**Claire Whittle,** Associate Dean, Academic Affairs and Business Development, Faculty of Education, Heart of England NHS Foundation Trust, Bordesley Green East, Birmingham B9 5SS, UK

## Figures and Tables

**Figure 1.**
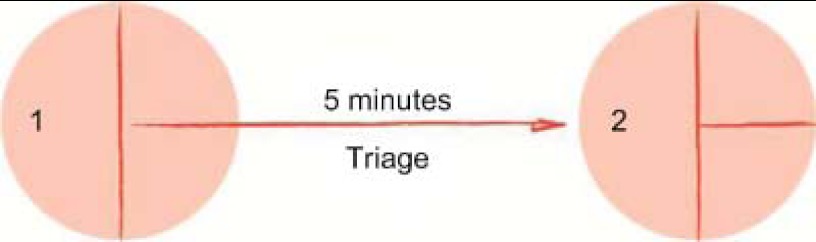
A simple flowchart.
